# Advances in Management of Voice and Swallowing Disorders

**DOI:** 10.3390/jcm11092308

**Published:** 2022-04-21

**Authors:** Renée Speyer

**Affiliations:** 1 Department Special Needs Education, Faculty of Educational Sciences, University of Oslo, 0318 Oslo, Norway; renee.speijer@isp.uio.no; 2 Curtin School of Allied Health, Faculty of Health Sciences, Curtin University, Perth, WA 6102, Australia; 3 Department of Otorhinolaryngology and Head and Neck Surgery, Leiden University Medical Centre, 2333 ZA Leiden, The Netherlands

Dysphagia (swallowing disorders) and dysphonia (voice disorders) are both common disorders within the area of laryngology. Recent research has focused on instrument development and psychometrics, and the development of methods with robust measurement properties (i.e., validity, reliability and responsiveness). In addition, newly developed interventions are waiting to be evaluated to objectify treatment effects. The outcomes of both instrument development and intervention studies will support evidence-based clinical practice and research [1]. This current Special Issue of the *Journal of Clinical Medicine* (*JCM*) describes both ongoing instrument development and intervention studies targeting people with dysphagia and dysphonia.

A reliability study by Kim et al. [2] confirmed that computer analysis using a deep learning model could detect laryngeal penetration or aspiration in recordings of videofluoroscopic swallowing studies (VFSS) as reliably as human examiners. These results provide further evidence to support the clinical application of deep learning technology in addition to the visuoperceptual evaluation of videofluoroscopic and possibly endoscopic recordings of swallowing. A second study on VFSS by Swan et al. [3] reported on the development of the Visuoperceptual Measure for Videofluoroscopic swallow studies (VMV). The authors piloted their newly developed measure to determine its validity and reliability using classical test theory analysis, informed by the consensus-based standards for the selection of health measurement instruments (COSMIN) guidelines [4]. The results are promising and validation will be continued using larger sample sizes and an item response theory paradigm approach.

Two studies refer to assessment in dysphonia. The study by Caffier et al. [5] determined the test–retest reliability of the nine-item Voice Handicap Index (VHI-9i), a self-reported questionnaire on the subjective impact of voice disorders on patients’ daily lives. The authors found high reliability and, as presented here, revised the VHI-9i severity levels based on receiver operating characteristic (ROC) curve analysis. The second study, by Nguyen et al. [6], used pitch discrimination as a key index of auditory perception, to discriminate between people with and without a voice disorder. The authors advocate the use of pitch discrimination testing during comprehensive voice assessment.

Three studies report on behavioural interventions in people with voice and swallowing problems. Madill et al. [7] describe the efficacy of active ingredients in the treatment of muscle-tension voice disorders, whereas Sinkiewicz et al. [8] present the results of a rehabilitation program for occupational voice disorders in teachers. A third study by Park et al. [9] on lingual strengthening training in older adults compares a new progressive resistance exercise with a conventional isometric tongue strengthening exercise. Two other intervention studies by Song et al. [10] and Novakovic et al. [11] report on CO_2_ laser microsurgery in patients with unilateral vocal fold cancer [10] and supraglottic botulinum toxin injection in laryngeal sensory dysfunction [11], respectively. All five of these intervention studies contribute to evidence-based clinical practice by objectifying the effects of distinct interventions in laryngology.

This Special Issue includes three more studies by Speyer et al. [12,13,14]: three systematic reviews and meta-analyses of interventions in people with oropharyngeal dysphagia. All three reviews use the same study methods. The reviews follow the PRISMA guidelines [15,16], and include the highest level of evidence only, thus excluding any other study designs except for randomised controlled trials. Two reviews report on neurostimulation: (1) pharyngeal and neuromuscular electrical stimulation; and (2) brain neurostimulation. Although describing promising results, protocol heterogeneity, potential moderators and inconsistent reporting of the methodology resulted in conservative generalisations and interpretations of the meta-analyses. Both reviews confirmed the need for further randomised controlled trials with larger population sizes using standard protocols and reporting guidelines as achieved by international consensus. The third review reports on behavioural interventions. Again, although behavioural interventions show promising effects in people with oropharyngeal dysphagia, due to high heterogeneity between studies, generalisations of meta-analyses must be interpreted with care.

In summary, the studies included in this Special Issue contribute to instrument development and psychometrics, and to objectifying the effects of interventions in the area of laryngology. Future studies will continue to contribute to evidence-based clinical practice and research.

## Data Availability

Not applicable.

## References

[1] Speyer R., Cordier R., Farneti D., Nascimento W., Pilz W., Verin E., Walshe M., Woisard V. (2022). White Paper by the European Society for Swallowing Disorders: Screening and Non-instrumental Assessment for Dysphagia in Adults. Dysphagia.

[2] Kim Y., Kim H.-I., Park G., Kim S., Choi S.-I., Lee S. (2021). Reliability of Machine and Human Examiners for Detection of Laryngeal Penetration or Aspiration in Videofluoroscopic Swallowing Studies. Clin. Med..

[3] Swan K., Speyer R., Scharitzer M., Farneti D., Brown T., Cordier R. (2022). A Visuoperceptual Measure for Videofluoroscopic Swallow Studies (VMV): A Pilot Study of Validity and Reliability in Adults with Dysphagia. J. Clin. Med..

[4] Mokkink L.B., Prinsen C.A., Bouter L.M., de Vet H.C., Terwee C.B. (2016). The COnsensus-based Standards for the selection of health Measurement INstruments (COSMIN) and how to select an outcome measurement instrument. Braz. J. Phys. Ther..

[5] Caffier F., Nawka T., Neumann K., Seipelt M., Caffier P.P. (2021). Validation and Classification of the 9-Item Voice Handicap Index (VHI-9i). J. Clin. Med..

[6] Nguyen D.D., Chacon A.M., Novakovic D., Hodges N.J., Carding P.N., Madill C. (2022). Pitch Discrimination Testing in Patients with a Voice Disorder. J. Clin. Med..

[7] Madill C., Chacon A., Kirby E., Novakovic D., Nguyen D.D. (2021). Active Ingredients of Voice Therapy for Muscle Tension Voice Disorders: A Retrospective Data Audit. J. Clin. Med..

[8] Sinkiewicz A., Garstecka A., Mackiewicz-Nartowicz H., Nawrocka L., Wojciechowska W., Szkiełkowska A. (2021). The Effectiveness of Rehabilitation of Occupational Voice Disorders in a Health Resort Hospital Environment. J. Clin. Med..

[9] Park J.W., Oh C.H., Choi B.U., Hong H.J., Park J.H., Kim T.Y., Cho Y.J. (2021). Effect of Progressive Head Extension Swallowing Exercise on Lingual Strength in the Elderly: A Randomized Controlled Trial. J. Clin. Med..

[10] Song W., Caffier F., Nawka T., Ermakova T., Martin A., Mürbe D., Caffier P.P. (2021). T1a Glottic Cancer: Advances in Vocal Outcome Assessment after Transoral CO_2_-Laser Microsurgery Using the VEM. J. Clin. Med..

[11] Novakovic D., Sheth M., Stewart T., Sandham K., Madill C., Chacon A., Nguyen D.D. (2021). Supraglottic Botulinum Toxin Improves Symptoms in Patients with Laryngeal Sensory Dysfunction Manifesting as Abnormal Throat Sensation and/or Chronic Refractory Cough. J. Clin. Med..

[12] Speyer R., Sutt A.L., Bergström L., Hamdy S., Heijnen B.J., Remijn L., Wilkes-Gillan S., Cordier R. (2022). Neurostimulation in People with Oropharyngeal Dysphagia: A Systematic Review and Meta-Analyses of Randomised Controlled Trials—Part I: Pharyngeal and Neuromuscular Electrical Stimulation. J. Clin. Med..

[13] Speyer R., Sutt A.L., Bergström L., Hamdy S., Pommée T., Balaguer M., Kaale A., Cordier R. (2022). Neurostimulation in People with Oropharyngeal Dysphagia: A Systematic Review and Meta-Analysis of Randomised Controlled Trials—Part II: Brain Neurostimulation. J. Clin. Med..

[14] Speyer R., Cordier R., Sutt A.L., Remijn L., Heijnen B.J., Balaguer M., Pommée T., McInerney M., Bergström L. (2022). Behavioural Interventions in People with Oropharyngeal Dysphagia: A Systematic Review and Meta-Analysis of Randomised Clinical Trials. J. Clin. Med..

[15] Page M.J., McKenzie J.E., Bossuyt P.M., Boutron I., Hoffmann T.C., Mulrow C.D., Shamseer L., Tetzlaff J.M., Akl E.A., Moher D. (2021). The PRISMA 2020 statement: An updated guideline for reporting systematic reviews. Int. J. Surg..

[16] Page M.J., Moher D., Bossuyt P.M., Boutron I., Hoffmann T.C., Mulrow C.D., Shamseer L., Tetzlaff J.M., Akl E.A., McKenzie J.E. (2021). PRISMA 2020 explanation and elaboration: Updated guidance and exemplars for reporting systematic reviews. BMJ.

